# Chenodeoxycholic Acid Improves Embryo Implantation and Metabolic Health through Modulating Gut Microbiota–Host Metabolites Interaction during Early Pregnancy

**DOI:** 10.3390/antiox13010008

**Published:** 2023-12-19

**Authors:** Meixia Chen, Ying Zhao, Haifeng Ji, Lu Li, Hui Liu, Sixin Wang, Dongyan Zhang, Jingdong Yin, Jing Wang, Xin Zhang

**Affiliations:** 1 Institute of Animal Husbandry and Veterinary Medicine, Beijing Academy of Agriculture and Forestry Sciences, Beijing 100097, China; chenmeixia@baafs.net.cn (M.C.); jihaifeng@baafs.net.cn (H.J.); lilu990445@163.com (L.L.); liuhui@baafs.net.cn (H.L.); wangsixin@baafs.net.cn (S.W.); zhangdongyan@baafs.net.cn (D.Z.); 2 Key Laboratory of Feed Biotechnology of the Ministry of Agriculture and Rural Affairs, Institute of Feed Research, Chinese Academy of Agricultural Sciences, Beijing 100081, China; yingzhaocaas@163.com; 3 College of Life Science and Food Engineering, Hebei University of Engineering, Handan 056038, China; 4 State Key Laboratory of Animal Nutrition and Feeding, College of Animal Science and Technology, China Agricultural University, Beijing 100193, China; yinjd@cau.edu.cn

**Keywords:** chenodeoxycholic acid, early pregnancy, embryo implantation, gut microbiota, metabolic health, metabolites

## Abstract

Fetus loss in early pregnancy is of major concern to both humans and animals, and this issue is largely influenced by embryo implantation. Chenodeoxycholic acid (CDCA), a primary bile acid, contributes to metabolic improvements and protects against intrahepatic cholestasis of pregnancy. However, the effect of CDCA on embryo implantation during early pregnancy has not been investigated. The present study demonstrated that CDCA administration during early pregnancy improved embryo implantation in sows and rats, thereby improving the pregnancy outcomes of sows. CDCA significantly reduced inflammation, oxidative stress, and insulin resistance. The metabolomics analysis indicated significant differences in the fecal metabolome, especially regarding the level of secondary bile acids, between the control and CDCA-treated sows. CDCA also influenced the serum metabolite profiles in sows, and the serum L-Histidine level was significantly correlated with the abundance of 19 differential fecal metabolites. Importantly, L-Histidine administration improved embryo implantation and metabolic health in rats during early pregnancy. Moreover, CDCA administration during early pregnancy also led to long-term metabolic improvements in sows. Our data indicated that CDCA improved embryo implantation by alleviating inflammation and oxidative stress, improving insulin sensitivity, and modulating the interaction between the gut microbiota and host metabolites. Therefore, CDCA intervention is a potential therapeutic strategy regarding embryo loss during pregnancy.

## 1. Introduction

In recent years, low fertility has become a major health concern due to the declining growth rate of the global population. Embryo loss during pregnancy leads to low fertility in humans and other mammals. It affects 15–20% of human pregnancies and 30–40% of mammalian pregnancies, with about 75% of losses happening during early pregnancy [1,2]. Therefore, reducing early embryo implantation failure is imperative for pregnancy success in mammals and humans.

Embryo implantation occurs within a limited period, namely, the “window of implantation”, and is coordinated by ovarian estrogen and progesterone [3]. For example, the progesterone level is gradually increased in the luteal phase of mice, accompanied by a peak of estrogen on day (d) 4 of pregnancy, while embryo implantation occurs at midnight on d 4 [4,5]. Oxidative stress has been linked to several reproductive problems, including abortions, pre-eclampsia, and gestational diabetes [6]. Our previous study also revealed the adverse effects of reactive oxygen species (ROS) overproduction on embryo implantation, using Ishikawa and JAR cells [7]. Therefore, it is of great importance to maintain oxidative and antioxidant balance during early pregnancy.

Embryo implantation is strongly influenced by maternal nutrition status. Non-enzymatic antioxidants, such as melatonin and resveratrol, have been used to improve embryo implantation and pregnancy outcomes through their antioxidative properties [8,9]. Additionally, maternal methionine consumption during early pregnancy increased the number of implantation sites in rats by promoting cell proliferation and DNA synthesis [10]. Zeng and co-workers also reported arginine as a promising candidate to enhance embryo implantation during early pregnancy by stimulating the activation of PI3K/PKB/mTOR/NO signaling [11]. Through the conduction of untargeted metabolomics in follicular fluid, urine, and serum, we also observed a disturbance of amino acid metabolism in sows exhibiting low reproductive performance [12]. To date, the effects of some essential amino acids, such as histidine, on embryo implantation have not been well explored.

Significantly, the gut microbiota is exquisitely sensitive to dietary factors, and a recent review pointed to a strong interaction between the gut microbiota and estrogen [13]. Accumulating evidence has demonstrated that the gut microbiota contributes to embryo implantation, embryo development, metabolic homeostasis, pregnancy outcomes, and even the health status of offspring through the production of metabolites, such as bile acids (BAs) and short-chain fatty acids [14,15,16,17]. However, how the gut microbiota and its derived metabolites promote embryo implantation in different maternal nutrition statuses is yet to be further elucidated.

CDCA is a primary BA that is synthesized from cholesterol in the liver and converted to secondary BAs by the gut microbiota. CDCA was reported to stimulate brown adipose tissue activity in humans [18] and to increase fatty acid oxidation in differentiated 3T3-L1 adipocytes [19], leading to metabolic improvements. Strikingly, CDCA treatment combated oxidative stress in porcine intestinal epithelial cells [20], implying its potential application as a natural antioxidant. As yet, no effects of CDCA on embryo implantation and pregnancy outcomes have been reported, but one study in rats revealed the protective role of CDCA in the development of intrahepatic cholestasis of pregnancy [21]. In the present study, pig and rat models were used to investigate the effects of dietary CDCA supplementation during early pregnancy on embryo implantation and pregnancy outcomes. Oxidative stress, inflammation, and metabolic landscapes were further analyzed. Additionally, the gut microbiota and fecal metabolites were analyzed during early pregnancy in sows. Our data revealed that CDCA treatment enhanced embryo implantation by means of an improvement in antioxidant capacity and metabolic homeostasis via modulating gut microbiota–serum metabolites interaction.

## 2. Materials and Methods

### 2.1. Experimental Animals and Sample Collection

All animal experiments were approved by the Animal Welfare and Ethical Committee of the Beijing Academy of Agriculture and Forestry Sciences (approval number: IHVM11-2202-2). A total of 24 Large White × Landrace-crossed sows (about 2.5 years old) were used in this study. After weaning, the sows were randomly divided into two groups according to parity, backfat thickness, and body weight (BW). Sows were housed in individual pregnancy stalls. Estrus was checked daily in the morning via direct boar exposure, and artificial insemination was conducted during estrus. Sows were fed basal diets or basal diets supplemented with 0.15% CDCA from weaning to early pregnancy (d 0 to 28 of pregnancy). The ingredient composition and nutrient level of basal diets are shown in Table S1 in the Supplementary Materials. After d 28 of pregnancy, all sows were fed the same diets. Sows had free access to clean drinking water throughout the experiment. The BW and backfat thickness of sows were measured at breeding, on d 14 of pregnancy, and on d 28 of pregnancy. At parturition, the litter size, live litter size, weight of piglets alive, and numbers of stillborn, deformed, and mummified piglets were recorded. On d 14 and d 110 of pregnancy, 10 mL blood samples were collected from the precaval vein and centrifuged at 3000× *g* for 15 min to obtain serum. The serum was stored at −80 °C until further analysis. Furthermore, fresh fecal samples were collected on d 14 of pregnancy using sterile 2-mL centrifuge tubes and then stored at −80 °C for gut microbiota and untargeted metabolomics analysis.

The effects of CDCA administration on embryo implantation were also determined in rats. Eight-week-old female Sprague Dawley rats were maintained under standard conditions (free access to food and water, in an environment with controlled temperature and humidity, and in 12 h of light and 12 h of darkness). After co-caging overnight, the presence of spermatozoa in the vaginal smear was defined as d 1 of pregnancy. To determine the effects of dietary CDCA supplementation on embryo implantation, the pregnant rats were fed control diets (according to AIN 93) or diets supplemented with 0.05%, 0.1%, or 0.5% CDCA (*n* = 12–14 per group). Similarly, to determine the effects of L-Histidine on embryo implantation, pregnant rats were fed control diets (according to AIN 93, with feed containing 0.4% L-Histidine) or diets plus 0.4% and 0.8% (wt:wt) L-Histidine (*n* = 11 per group), respectively. On d 7 of pregnancy, the rats were anesthetized by an intravenous injection of 2.0% sodium pentobarbital (30 mg/kg BW) and then sacrificed for serum collection. The uterine horns were immediately exposed to record the number of implantation sites.

### 2.2. Feed Composition Analysis

The basal diets of the sows were analyzed for crude protein, according to AOAC [22]. The amino acid composition was determined, as described previously [23]. Except for methionine, cysteine, and tryptophan, the amino acids were hydrolyzed with 6 mol/L HCl, and the levels were determined using an amino acid analyzer (Hitachi L-8900, Tokyo, Japan). The methionine and cysteine levels were determined using an amino acid analyzer (Hitachi L-8900, Tokyo, Japan) after cold performic acid oxidation overnight and hydrolyzing with 7.5 mol/L HCl at 110 °C for 24 h. After alkaline hydrolysis (LiOH) for 22 h at 110 °C, the tryptophan level was determined via high-performance liquid chromatography (Agilent 1200 Series, Santa Clara, CA, USA).

### 2.3. Measurement of Serum Biochemical Indicators

Serum estradiol and progesterone levels were measured via radioimmunoassay (Sinoukbio, Beijing, China). The activities of total antioxidant capacity (T-AOC), superoxide dismutase (SOD), catalase (CAT), glutathione peroxidase (GSH-Px), and the contents of malondialdehyde (MDA) in the serum were determined by commercial kits (Nanjing Jiancheng Bioengineering Institute, Nanjing, China). Furthermore, the levels of tumor necrosis factor-α (TNF-α), interferon-γ (IFN-γ), interleukin-1β (IL1β), and IL6 were detected in the serum of sows and rats using ELISA kits (Nanjing Jiancheng Bioengineering Institute, Nanjing, China). To analyze glucose and lipid metabolism in sows, the concentrations of glucose, insulin, total cholesterol (TC), HDL-C, and LDL-C were ascertained. The homeostatic model assessment of insulin resistance (HOMA-IR) was calculated using the following equation: HOMA-IR = insulin (mIU·L^−1^) × glucose (mmol·L^−1^)/22.5.

### 2.4. Fatty Acid Profiles in Serum

To analyze the fatty acid profile in the serum of sows, samples (200 μL) were transferred to 1.5-mL EP tubes, extracted with 1 mL dichloromethane/methanol (1:1), and treated with 0.5 mL n-hexane. After being vortexed for 30 s, the mixtures were centrifuged at 13,000× *g* for 10 min. The supernatant was analyzed via gas chromatography (Agilent 8890B-7000D, Agilent Technologies Inc., Santa Clara, CA, USA), following the manufacturer’s instructions for a colorimetric assay.

### 2.5. Untargeted Metabolomics and Data Analysis

Metabolite concentrations in the feces and serum of sows were determined by UHPLC-MS/MS in both positive and negative ionization modes. Fecal samples of 50 mg and 100 μL serum samples were used, respectively. The samples were mixed with a 400 µL methanol:water (4:1, *v*/*v*) solution, in which 0.02 mg/mL L-2-chlorophenylalanine was used as the internal standard. The mixtures were sonicated for 30 min at low temperatures and centrifuged at 13,000× *g* for 15 min at 4 °C. The supernatant containing metabolites was dried, dissolved in 100 µL acetonitrile:water (1:1 *v*/*v*), and transferred to a sample vial for LC-MS/MS analysis using a Thermo UHPLC-Q Exactive HF-X system. Two μL of the sample was separated via an HSS T3 column (2.1 × 100 mm, 1.8 μm) at 40 °C at a flow rate of 0.4 mL/min. The data were preprocessed and annotated using HMDB and Metlin, as described previously [24]. The data were applied for principal component analysis (PCA) and partial least squares discriminant analysis (PLS-DA). Metabolites with a variable importance projection (VIP) of >1.0 and *p* < 0.05 were significantly differential metabolites. KEGG enrichment analysis (http://www.genome.jp/kegg/ (accessed on 20 October 2022)) was also performed to interpret the biological significance of metabolites.

### 2.6. BAs Quantification in Fecal Samples

UPLC, coupled with a QTRAP^®^ 6500+ mass spectrometer (AB SCIEX, Framingham, MA, USA), was used to analyze the BAs. Briefly, 50 mg fecal samples were extracted with methanol containing deuterated internal standards. After vortex and centrifugation at 13,000× *g* for 15 min, the supernatant was evaporated and reconstituted with 50% acetonitrile. The BAs were separated via a C18 column (1.7 µm, 2.1 × 150 mm), with water and acetonitrile as the mobile phases, and were detected using MRM in negative mode. Data processing was performed using Analyst (AB SCIEX, Framingham, MA, USA).

### 2.7. DNA Extraction, 16S rRNA Sequencing, and Data Analysis

The microbial community genomic DNA in fresh feces was extracted using the Mag-Bind^®^ Soil DNA Kit (M5636, Omega, Norcross, GA, USA). DNA quality was accessed via agarose gel electrophoresis and its concentration was determined by the spectrophotometer. PCR was performed to amplify the V3 + V4 regions of the 16S rRNA genes using the primer pairs (Forward: ACTCCTACGGGAGGCAGCAG, Reverse: GGACTACHVGGGTWTCTAAT). The PCR product was extracted from 2% agarose gel and purified using an AxyPrep DNA Gel Extraction Kit (AP-GX-50, Axygen Biosciences, Union City, CA, USA), following the manufacturer’s instructions. Purified amplicons were pooled in equimolar amounts and subjected to paired-end sequencing on an Illumina MiSeq PE300 platform/NovaSeq PE250 platform (Illumina, San Diego, CA, USA), according to the standard protocols of Majorbio Bio-Pharm Technology Co., Ltd. (Shanghai, China).

The DADA2 plug-in in QIIME2 software (https://qiime2.org/ (accessed on 6 February 2023)) was used to filter, denoise, merge, and remove non-chimeric sequences from all raw sequences to form operational taxonomic units (OTUs). All OTUs were then subjected to cluster analysis and taxonomic analysis. Representative sequences from the OTUs were aligned with the Silva Release 138 database to obtain the annotation information regarding the species. Then, the α-diversity index, β-diversity index, and species abundances were analyzed separately for each taxonomic level. Various α-diversity indexes, such as the Shannon index, Simpson index, Chao1 richness estimator, and abundance-based coverage estimator (ACE) metric, were calculated using QIIME (version 1.9.1). The linear discriminant analysis (LDA) effect size (LEfSe) (http://huttenhower.sph.harvard.edu/LEfSe (accessed on 6 February 2023)) was used to identify bacterial taxa (phylum to genera) that were significantly abundant in the different groups (LDA score > 2, *p* < 0.05).

### 2.8. Statistical Analysis

Data was expressed as means ± SEM. Statistical significance was analyzed using an unpaired two-tailed Student’s *t*-test or the one-way ANOVA procedures of SAS (v.9.2, SAS Institute, Cary, NC, USA), except where indicated. The relationships between key parameters were assessed via Spearman’s correlation. A Procrustes analysis for fecal metabolites and serum metabolites was performed using the Procrustes function in the vegan R package. A value of *p* < 0.05 was considered significant, and 0.05 ≤ *p* ≤ 0.10 was considered to indicate a trend.

## 3. Results

### 3.1. CDCA Improves Embryo Implantation and Pregnancy Outcomes

As shown in Table 1, the dietary supplementation of 0.15% CDCA during estrus and early pregnancy had no effect on BW and the backfat thickness of sows on d 14 and d 28 of pregnancy. CDCA supplementation markedly increased the number of total piglets and live-born piglets (*p* < 0.05, Table 2) compared with the number in the control, while no obvious differences were observed in terms of BW, sex ratio, stillbirth occurrence, deformation, and mummification (Table 2).

**Figure 1:**
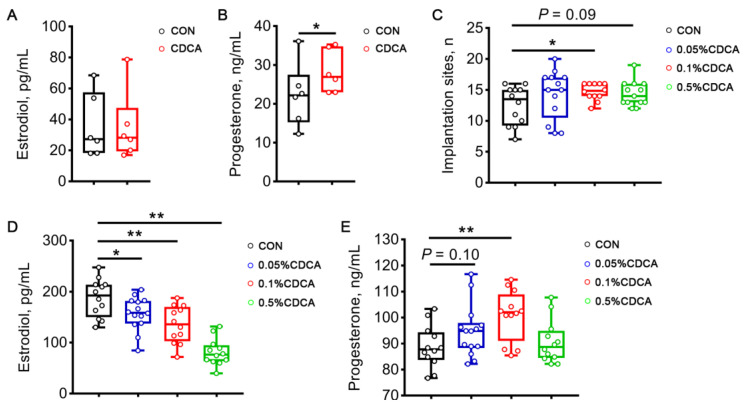
Effects of CDCA supplementation on sex hormone synthesis and embryo implantation. (**A**,**B**) Concentrations of estradiol and progesterone in the serum of sows on d 14 of pregnancy. (**C**) The number of implantation sites in rats on d 7 of pregnancy. (**D**,**E**) Concentrations of estradiol and progesterone in the serum of rats on d 7 of pregnancy. Data are presented as means ± SEM. *n* = 6 for (**A**,**B**), and *n* = 12–13 for (**C**–**E**). Statistical significance was ascertained with Student’s *t*-test or a one-way ANOVA. * *p* < 0.05, ** *p* < 0.01 compared with the control group.

To determine the role of CDCA in embryo implantation, the concentrations of estradiol and progesterone in the serum of sows were determined on d 14 of pregnancy. The results showed that CDCA treatment significantly increased the level of progesterone (*p* < 0.05, Figure 1B) but had no effect on the level of estradiol (Figure 1A).

It is difficult and expensive to detect embryo implantation in sows. Therefore, Sprague Dawley rats were further used to explore the beneficial effect of CDCA on embryo implantation during early pregnancy. It transpired that diets containing 0.1% CDCA significantly increased the number of implantation sites (*p* < 0.05, Figure 1C), decreased the concentration of estradiol (*p* < 0.01, Figure 1D), and elevated the concentration of progesterone (*p* < 0.01, Figure 1E) on d 7 of pregnancy in rats. Collectively, these results suggest that the dietary supplementation of CDCA had a positive influence on embryo implantation during early pregnancy, which contributed greatly to improved pregnancy outcomes.

### 3.2. CDCA Alleviates Inflammation and Enhances Antioxidant Capacity during Early Pregnancy

**Figure 2:**
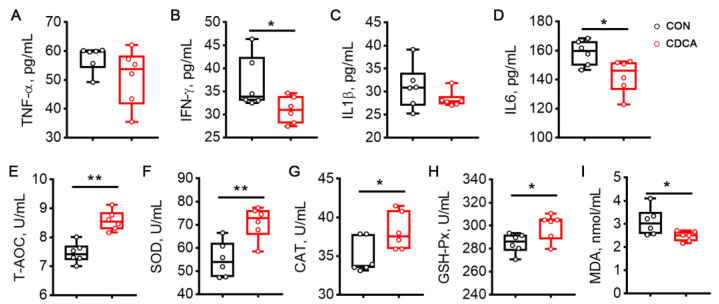
Effects of CDCA supplementation on systemic inflammation and oxidative stress in sows. The serum levels of TNF-α (**A**), IFN-γ (**B**), IL1β (**C**), and IL6 (**D**) were measured on d 14 of pregnancy. The activities of T-AOC (**E**), SOD (**F**), CAT (**G**), and GSH-Px (**H**), as well as the content of MDA (**I**) were also determined in the serum on d 14 of pregnancy. Data are presented as means ± SEM (*n* = 6). Statistical significance was ascertained with Student’s *t*-test. * *p* < 0.05, ** *p* < 0.01.

Given the beneficial effect of CDCA on embryo implantation, we next conducted a systemic evaluation of inflammation and antioxidant capacity during early pregnancy in sows. We found that CDCA significantly decreased the contents of IFN-γ and IL6 in the serum on d 14 of pregnancy (*p* < 0.05, Figure 2B,D). It should be noted that CDCA also elevated the activities of T-AOC, SOD, CAT, and GSH-Px, and decreased the concentration of MDA in the serum of sows (Figure 2E–I).

**Figure 3:**
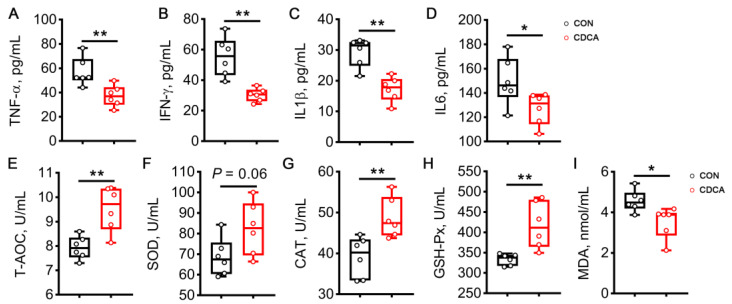
Effects of 0.1% CDCA administration on systemic inflammation and oxidative stress in rats. The serum levels of TNF-α (**A**), IFN-γ (**B**), IL1β (**C**), and IL6 (**D**) were measured on d 7 of pregnancy. The activities of T-AOC (**E**), SOD (**F**), CAT (**G**), and GSH-Px (**H**), as well as the content of MDA (**I**) were also determined in the serum on d 7 of pregnancy. Data are presented as means ± SEM (*n* = 6). Statistical significance was ascertained with Student’s *t*-test. * *p* < 0.05, ** *p* < 0.01.

The protective role of CDCA in combating inflammation and oxidative stress was also determined in rats. Dietary supplementation of 0.1% CDCA decreased the levels of inflammatory factors on d 7 of pregnancy in rats, including TNF-α, IFN-γ, IL1β, and IL6 (Figure 3A–D). CDCA treatment also increased the activities of T-AOC, CAT, and GSH-Px, and decreased the MDA content in the serum of rats (Figure 3E–I).

### 3.3. CDCA Confers Protection against Insulin Resistance and Reduces Lipid Accumulation

**Figure 4:**
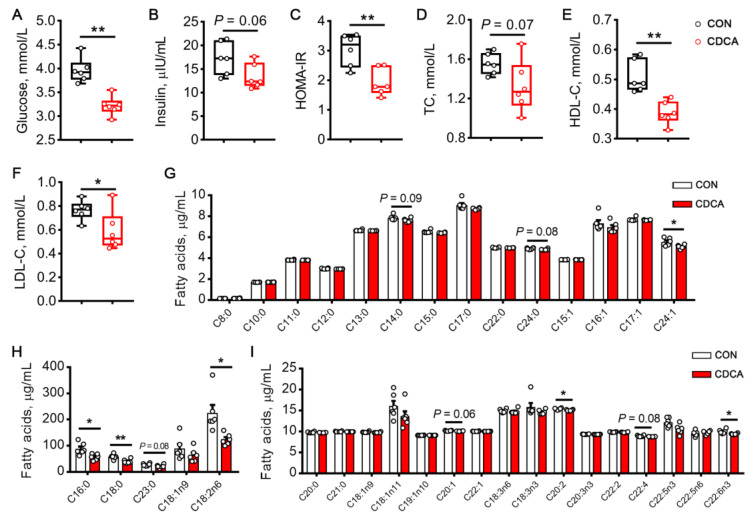
Effects of CDCA supplementation on glucose and lipid metabolism in sows. After fasting for 12 h, the blood glucose (**A**) and serum insulin (**B**) levels were measured on d 14 of pregnancy. (**C**) The HOMA-IR index was calculated, based on glucose and insulin levels. The TC (**D**), HDL-C (**E**), LDL-C (**F**) levels, and fatty acids profiles (**G**–**I**) were examined in the serum. Data are presented as means ± SEM (*n* = 6). Statistical significance was ascertained with Student’s *t*-test. * *p* < 0.05, ** *p* < 0.01.

To explore the function of CDCA in metabolic health during embryo implantation, we tested the glucose and insulin levels in the serum of sows on d 14 of pregnancy. CDCA treatment significantly decreased glucose levels (*p* < 0.01, Figure 4A) and tended to decrease insulin levels (*p* = 0.06, Figure 4B). Consequently, HOMA-IR was significantly decreased in sows from the CDCA group (*p* < 0.01, Figure 4C). Consistent with enhanced insulin sensitivity, CDCA supplementation led to a reduction in TC (*p* = 0.07, Figure 4D), HDL-C (*p* < 0.01, Figure 4E), and LDL-C levels (*p* < 0.05, Figure 4F). Conversely, as expected, some species of saturated fatty acids (C14:0, C16:0, C18:0, C23:0, and C24:0), monounsaturated fatty acids (C20:1 and C24:1), and polyunsaturated fatty acids (C18:2n6, C20:2, C22:4, and C22:6n3) were all downregulated in the serum of the CDCA group (Figure 4G–I). These data indicated that CDCA treatment during early pregnancy may improve glucose and lipid metabolism and, thus, improve metabolic health.

### 3.4. CDCA Has Little Effect on the Gut Microbiota Composition in Sows

**Figure 5:**
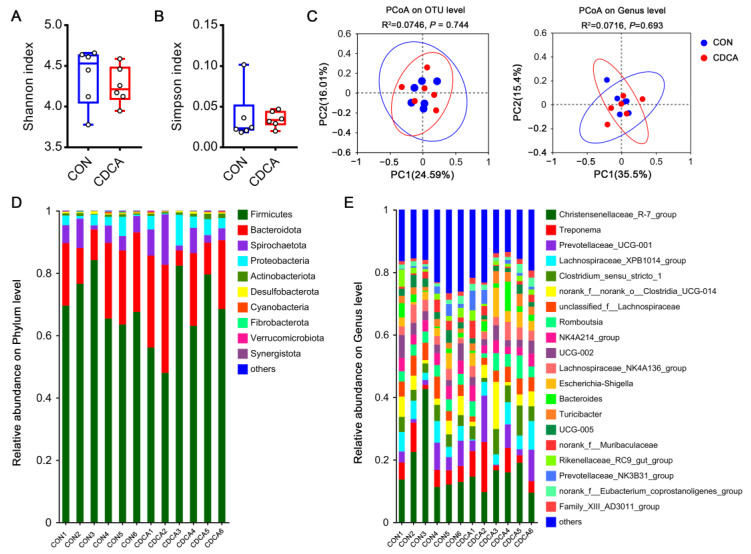
Effects of CDCA supplementation on gut microbiota composition in sows. An analysis of the gut microbiota was performed using fecal samples, which were collected on d 14 of pregnancy. The Shannon index (**A**) and Simpson index (**B**) were used to describe the α-diversity of gut microbiota. (**C**) Unweighted principal coordinate analysis (PCoA) was used to access the β-diversity at the OTU and genus levels, respectively. The relative abundance of bacteria is shown at phylum (**D**) and genus (**E**) levels. The top 10 most abundant phyla and top 20 most abundant genera are shown. Data are presented as means ± SEM (*n* = 6). Statistical significance was ascertained with Student’s *t*-test.

The gut microbiota is considered to be an endocrine organ that influences the reproductive endocrine system by interacting with estrogen, insulin, and other hormones [13]. In this study, the influence of CDCA treatment on gut microbiota was explored on d 14 of pregnancy in sows. Compared with the control group, the α-diversity of the gut microbiota was not influenced by CDCA treatment, as evidenced by the unchanged Shannon index and Simpson index (Figure 5A,B). Next, unweighted principal coordinate analysis (PCoA) was used to access the β-diversity of the gut microbiota. PCoA on OTU and genus levels revealed that the overall gut microbiota composition in the two groups was not significantly different (Figure 5C). At the phylum level, Firmicutes and Bacteroidota were the dominant phyla in the control and CDCA groups (Figure 5D). At the genus level, *Christensenellaceae_R-7_group* and *Treponema* were the dominant genera (Figure 5E).

### 3.5. CDCA Reshapes the Fecal Metabolome during Early Pregnancy

**Figure 6:**
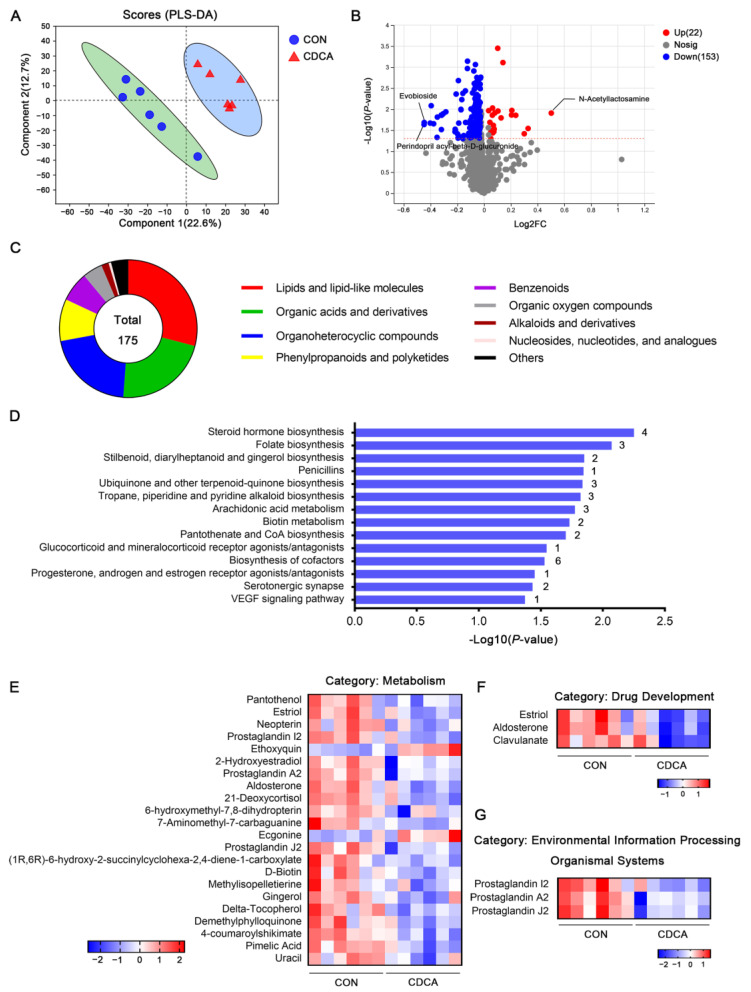
Effects of CDCA supplementation on fecal metabolome in sows. The untargeted metabolomics profile of fecal samples was analyzed on d 14 of pregnancy (*n* = 6). (**A**) PLS-DA showed a separation of data, depending on CDCA supplementation. (**B**) Volcano plots of all metabolites. Red modes and blue modes represent the upregulated and downregulated metabolites in the CDCA group, respectively. The dotted horizontal line indicates the threshold for a *p*-value of 0.05. (**C**) The classification of total differential metabolites, based on HMDB database annotation. (**D**) KEGG enrichment analysis was performed, based on the differential metabolites. The number on the right of the columns represents the number of metabolites enriched in each KEGG pathway. Heatmap of the metabolites involved in four KEGG pathway categories, including metabolism (**E**), drug development (**F**), environmental information processing, and organismal systems (**G**).

The gut microbiota has been reported to modulate host metabolism through the production of a large number of metabolites. Therefore, we next compared fecal untargeted metabolomics profiles between the two groups of sows that were fed control and CDCA diets on d 14 of pregnancy. PLS-DA corroborated the presence of a clear separation between the two groups (Figure 6A). Under the conditions of *p* < 0.05 and VIP > 1.0, 22 metabolites were significantly upregulated and 153 metabolites were downregulated in the CDCA group (Table S2 in the Supplementary Materials). N-acetyllactosamine (N-Lac) was the most upregulated metabolite. Meanwhile, perindopril acyl-beta-D-glucuronide and evobioside were the top two downregulated metabolites (Figure 6B). According to the HMDB database annotation, 175 differential metabolites belonged to 9 classes. Most metabolites were lipids, organic acids and their derivatives, and organoheterocyclic compounds (Figure 6C). KEGG enrichment analysis further revealed that differential metabolites were involved in the pathways associated with metabolism, drug development, environmental information processing, and the organismal system (Figure 6D). In brief, lipid metabolism (steroid hormone biosynthesis; arachidonic acid metabolism), vitamin metabolism (folate biosynthesis; ubiquinone and other terpenoid–quinone biosynthesis; biotin metabolism; pantothenate and CoA biosynthesis), the biosynthesis of cofactors, and the biosynthesis of other secondary metabolites (stilbenoid, diarylheptanoid, and gingerol biosynthesis; tropane, piperidine, and pyridine alkaloid biosynthesis) were the significantly enriched metabolic pathways. A total of 22 metabolites were involved in the above pathways; notably, most of them were downregulated in the CDCA group, except for ethoxyquin and ecgonine (Figure 6E). Additionally, the metabolites involved in drug development (glucocorticoid and mineralocorticoid receptor agonists/antagonists; progesterone, androgen, and estrogen receptor agonists/antagonists; penicillins) were downregulated in the CDCA group, including estriol, aldosterone, and clavulanate (Figure 6F). More interestingly, the levels of prostaglandins I2, A2, and J2 were all decreased in the CDCA group, which were enriched in the VEGF signaling and serotonergic synapse pathways (Figure 6G).

**Figure 7:**
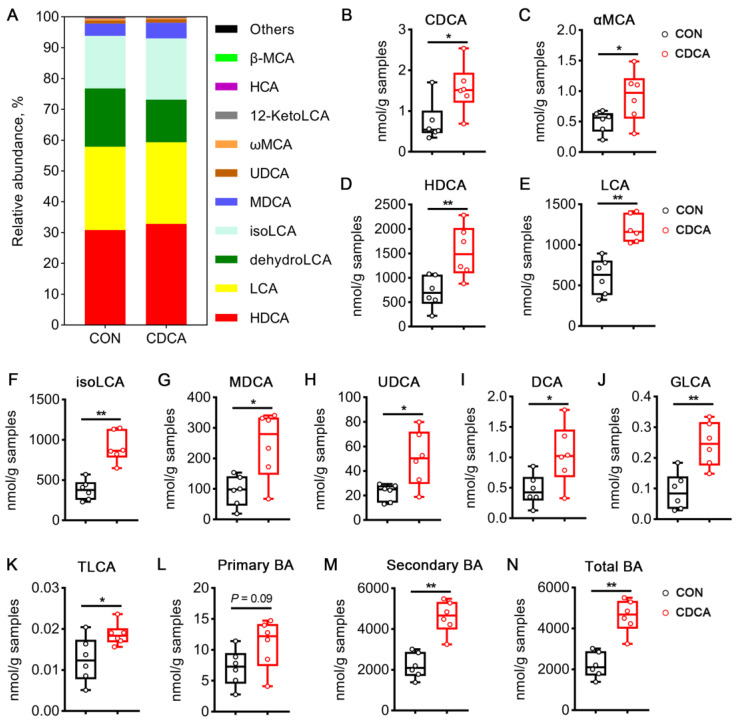
Effects of CDCA supplementation on bile acid (BA) profiles in the feces of sows. (**A**) Relative abundances of BAs in feces. (**B**–**K**) The concentrations of CDCA, αMCA, HDCA, LCA, isoLCA, MDCA, UDCA, DCA, GLCA, and TLCA. (**L**–**N**) The concentrations of primary BAs, secondary BAs, and total BAs. Data are presented as means ± SEM (*n* = 6). Statistical significance was ascertained with Student’s *t*-test. * *p* < 0.05, ** *p* < 0.01.

To further investigate the metabolic alterations by CDCA treatment, we conducted targeted metabolomics profiling of BAs in fecal samples. Hyodeoxycholic acid (HDCA), lithocholic acid (LCA), dehydrolithocholic acid (dehydroLCA), and isolithocholic acid (isoLCA) were the major BAs in the feces of sows, as can be observed from the results of relative abundance (Figure 7A). The primary BAs, including CDCA and alpha-muricholic acid (αMCA), were significantly increased in the feces of CDCA-treated sows (Figure 7B,C). As shown in Figure 7D–K, CDCA administration also increased the contents of eight secondary BAs, including HDCA, LCA, isoLCA, murideoxycholic acid (MDCA), ursodeoxycholic acid (UDCA), deoxycholic acid (DCA), glycolithocholic acid (GLCA), and taurolithocholic acid (TLCA). As a consequence, on the whole, the levels of secondary BAs and total BAs were significantly increased in the CDCA group (Figure 7M,N). Therefore, our data suggest that CDCA altered BA metabolism in sows during early pregnancy.

### 3.6. Associations between Fecal Metabolites and Host Metabolites

**Figure 8:**
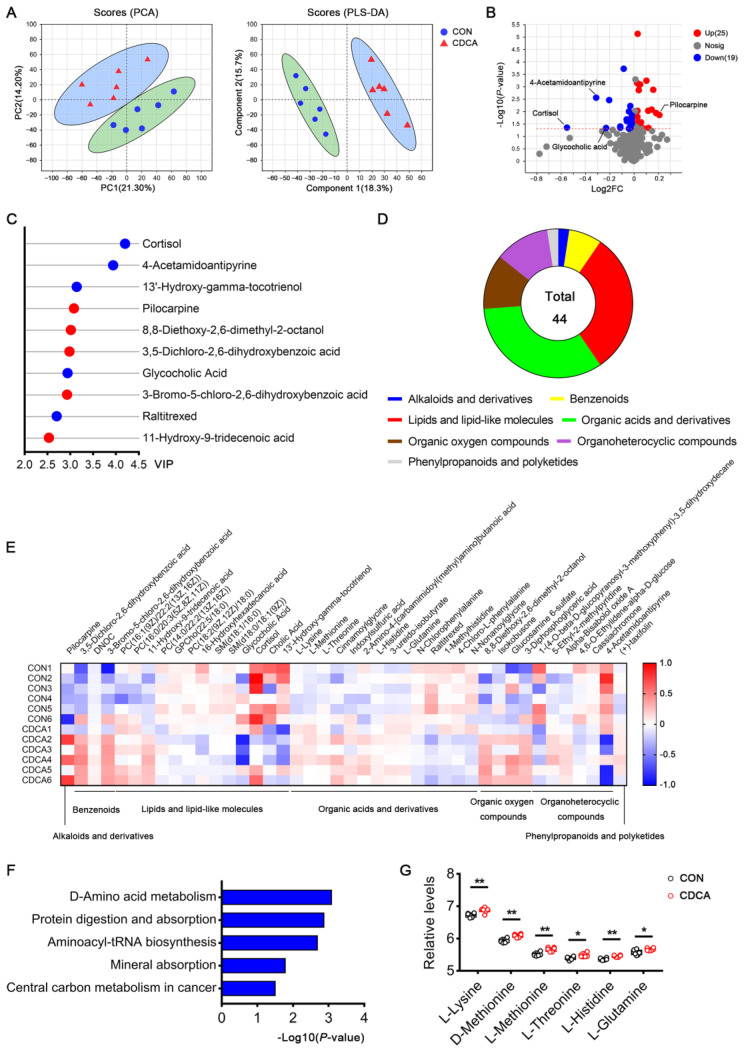
Effects of CDCA supplementation on serum metabolomics profiles in sows. Untargeted metabolomics approaches were employed to measure the serum metabolites on d 14 of pregnancy (*n* = 6). (**A**) Principal component analysis (PCA) and partial least squares discriminant analysis (PLS-DA) showed a separation of data, depending on CDCA supplementation. (**B**) Volcano plots of all metabolites. The red modes and blue modes represent the upregulated and downregulated metabolites in the CDCA group, respectively. The dotted horizontal line indicates the threshold for a *p*-value of 0.05. (**C**) VIP scores showing the top 10 metabolites responsible for the data separation. The red modes and blue modes represent the upregulated and downregulated metabolites in the CDCA group, respectively. (**D**) The classification of total differential metabolites, based on HMDB database annotation. (**E**) A heat map of the hierarchical clustering analysis for the two groups is presented. (**F**) Enrichment analysis of differential metabolites, based on the KEGG database. (**G**) The relative levels of the differential metabolites enriched in the KEGG pathways are shown in (**F**). Data are presented as means ± SEM. * *p* < 0.05, ** *p* < 0.01.

Untargeted metabolomics approaches were employed to detect the serum metabolites on d 14 of pregnancy in sows. PCA and PLS-DA exhibited a clear separation between the control and CDCA groups (Figure 8A). Briefly, a total of 485 metabolites were detected. Among them, 25 metabolites were upregulated, while 19 were downregulated in the CDCA group (Figure 8B). The top 10 metabolites responsible for the greatest separation of the data were also revealed using VIP scores (Figure 8C). According to the VIP scores, cortisol, 4-acetamidoantipyrine, and 13′-hydroxy-gamma-tocotrienol were the top three downregulated metabolites (blue nodes in Figure 8C). Pilocarpine, 3-bromo-5-chloro-2,6-dihydroxybenzoic acid, and 8,8-diethoxy-2,6-dimethyl-2-octanol were the top three upregulated metabolites (red nodes in Figure 8C). Interestingly, among these 44 metabolites, 14 were organic acids and derivatives, and 13 were lipids and lipid-like molecules (Figure 8D). The relative abundances of differential metabolites were listed in Figure 8E, which demonstrated that the abundances of nine metabolites belonging to lipids and lipid-like molecules had decreased in the CDCA group, such as cortisol, glycocholic acid, and cholic acid. More importantly, the abundances of amino acids, including L-Lysine, L-Methionine, L-Threonine, L-Histidine, and L-Glutamine, were elevated by CDCA supplementation. In contrast, the abundances of amino acid derivatives, such as N-Chlorophenylalanine, 1-Methylhistidine, 4-Chloro-L-phenylalanine, and N-Nonanoylglycine were decreased in the CDCA group. In accordance with the dramatically changed amino acids profile, D-amino acid metabolism and protein digestion and absorption were highly enriched within the range of amino acids exclusively upregulated in the CDCA group (Figure 8F,G). These results highlight the important role of amino acids in the beneficial effects of CDCA during early pregnancy.

**Figure 9:**
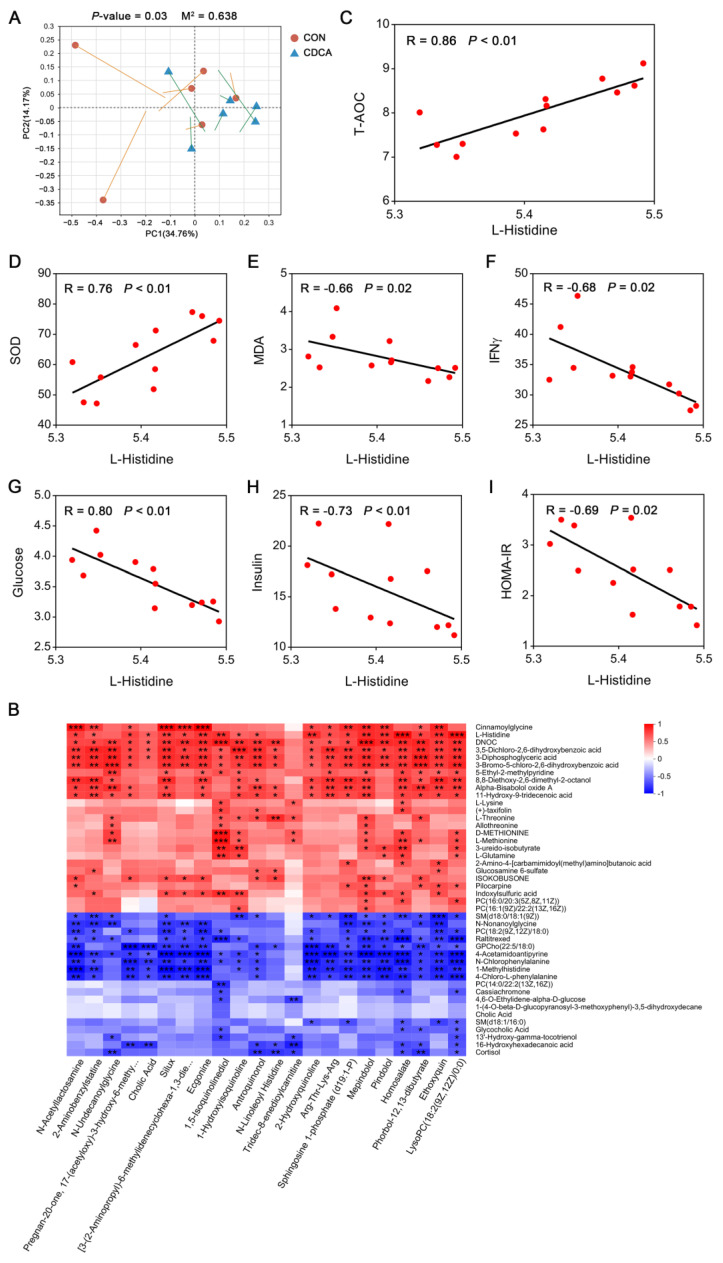
Associations between fecal metabolites and circulating metabolites. (**A**) Procrustes analysis for the correlations between the fecal metabolome and circulating metabolites in sows. (**B**) Spearman’s rank correlation between 22 upregulated metabolites in the fecal samples and 44 different metabolites in the serum. The red cells and blue cells represent positive and negative correlations, respectively. * *p* < 0.05, ** *p* < 0.01, *** *p* < 0.001. The scatter plot indicates the Spearman’s correlation coefficient, with statistical significance (*p* < 0.05) between serum L-Histidine levels and T-AOC (**C**), SOD (**D**), MDA (**E**), IFNγ (**F**), glucose (**G**), insulin (**H**), and HOMA-IR (**I**).

The link between the abundance of circulating metabolites and fecal metabolites was investigated. A significant relatedness (*p* = 0.03, M^2^ = 0.638) between the fecal metabolome and serum metabolome was established via Procrustes analysis (Figure 9A). Spearman analysis was next performed to test the association between 44 circulating metabolites and 22 upregulated fecal metabolites (Figure 9B). DNOC, 3,5-Dichloro-2,6-dihydroxybenzoic acid, 3-Diphosphoglyceric acid, and 3-Bromo-5-chloro-2,6-dihydroxybenzoic acid were significantly correlated with 21 fecal metabolites. L-Histidine was significantly correlated with 19 fecal metabolites. Moreover, the correlation between serum L-Histidine and antioxidant capacity was further visualized using a scatter plot. The results showed that the increased activities of T-AOC and SOD, and the decreased content of MDA, were associated with higher L-Histidine levels in sows (Figure 9C–E). A negative correlation between the level of IFN-γ and the level of L-Histidine was also observed (Figure 9F). Additionally, the increased insulin sensitivity was positively associated with higher levels of circulating L-Histidine in sows (Figure 9G–I).

### 3.7. L-Histidine Enhances Embryo Implantation and Attenuates Inflammation, Oxidative Stress, and Insulin Resistance during Early Pregnancy

**Figure 10:**
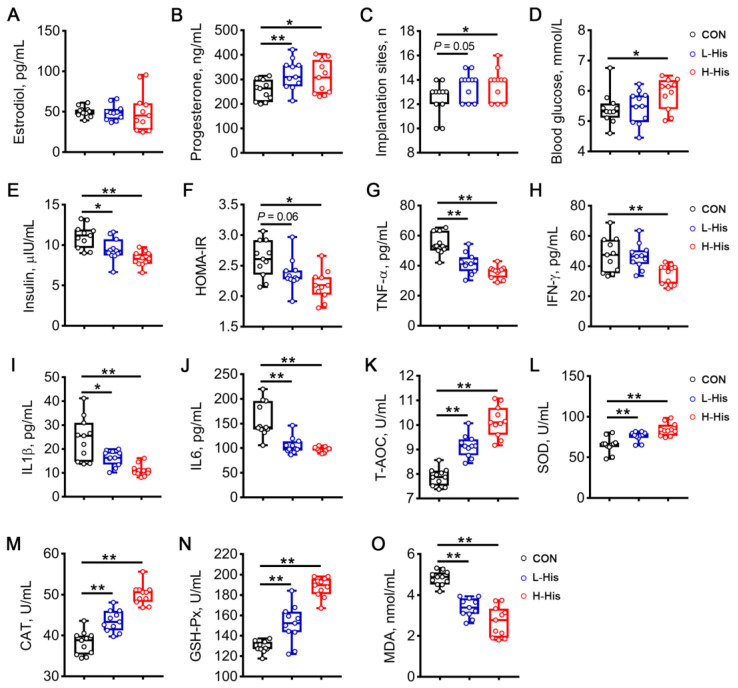
Effects of L-Histidine administration on embryo implantation and metabolic health during early pregnancy in rats. Pregnant rats were fed control diets (containing 0.4% L-Histidine) or diets plus 0.4% (L-His) and 0.8% L-Histidine (H-His). The serum levels of estradiol (**A**) and progesterone (**B**) and the number of implantation sites (**C**) were measured on d 7 of pregnancy. The levels of blood glucose (**D**) and insulin (**E**) were determined and HOMA-IR (**F**) was calculated. Serum samples were also used for the measurement of TNF-α (**G**), IFN-γ (**H**), IL1β (**I**), and IL6 (**J**) levels, T-AOC (**K**), SOD (**L**), CAT (**M**), and GSH-Px (**N**) activities, and MDA contents (**O**). Data are presented as means ± SEM (*n* = 11). Statistical significance was ascertained with Student’s *t*-test. * *p* < 0.05, ** *p* < 0.01.

To determine whether L-Histidine was involved in the beneficial effects of CDCA on embryo implantation, rats were administered 0.4% (CON), 0.8%, and 1.2% L-Histidine during early pregnancy. The results showed that compared with the control diet, a diet containing 1.2% L-Histidine increased the concentration of progesterone (*p* < 0.05, Figure 10B) and the number of implantation sites (*p* < 0.05, Figure 10C). Additionally, compared with the control diet, the diet containing 1.2% L-Histidine not only decreased the insulin levels (*p* < 0.01, Figure 10E) and HOMA-IR (*p* < 0.05, Figure 10F) but also increased the glucose levels (*p* < 0.05, Figure 10D). Moreover, inflammation was attenuated by diets containing 0.8% and 1.2% L-Histidine, as evidenced by the decreased levels of TNF-α, IFN-γ, IL1β, and IL6 (Figure 10G–J). L-Histidine administration also significantly elevated the activities of T-AOC, SOD, CAT, and GSH-Px, whereas it decreased the MDA content in the serum (Figure 10K–O). Collectively, these observations suggest that proper dietary L-Histidine levels benefit embryo implantation during early implantation.

### 3.8. The Long-Term Beneficial Effects of CDCA Treatment during Early Pregnancy

**Figure 11:**
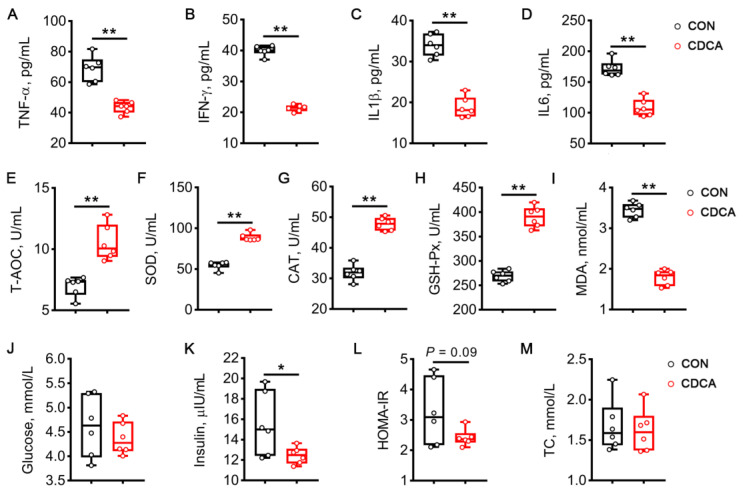
Long-term beneficial effects of CDCA supplementation during early pregnancy on systemic inflammation, oxidative stress, and insulin sensitivity in sows. The serum levels of TNF-α (**A**), IFN-γ (**B**), IL1β (**C**), and IL6 (**D**) were measured on d 110 of pregnancy. The activities of T-AOC (**E**), SOD (**F**), CAT (**G**), and GSH-Px (**H**), as well as the content of MDA (**I**), were also determined in the serum on d 110 of pregnancy. With regard to insulin sensitivity, the levels of blood glucose (**J**), serum insulin (**K**), and TC (**M**) were measured. (**L**) The HOMA-IR index was also calculated. Data are presented as means ± SEM (*n* = 6). Statistical significance was ascertained with Student’s *t*-test. * *p* < 0.05, ** *p* < 0.01.

The inflammation, antioxidant capacity, and glucose metabolism were detected on d 110 of pregnancy in sows to examine the long-term beneficial effects of CDCA treatment during early pregnancy. As shown in Figure 11A–D, dietary supplementation of 0.15% CDCA during early pregnancy dramatically decreased the levels of TNF-α, IFN-γ, IL1β, and IL6 in the serum on d 110 of pregnancy (*p* < 0.01). The increased activities of T-AOC, SOD, CAT, and GSH-Px were also observed in the CDCA group (*p* < 0.01, Figure 11E–H), and, consistently, CDCA treatment decreased the MDA contents in the serum (*p* < 0.01, Figure 11I). In terms of glucose metabolism, CDCA treatment significantly decreased the insulin levels (*p* < 0.05, Figure 11K), and tended to decrease HOMA-IR (*p* = 0.09, Figure 11L). These findings strongly indicate that dietary CDCA supplementation during early pregnancy contributes to long-term metabolic improvements in sows.

## 4. Discussion

During pregnancy, the anatomy and physiology of the pregnant mother undergo dramatic changes in order to nurture and adapt to the developing fetus. Increased insulin sensitivity occurs in early pregnancy, followed by progressive insulin resistance, increased lipid synthesis, and decreased protein catabolism [25]. Serum BA levels also show a progressive rise along with advancing gestation [26]. Here, CDCA supplementation during early pregnancy improved embryo implantation and pregnancy outcomes by ameliorating oxidative stress and inflammation, as well as enhancing insulin sensitivity in pig and rat models. Interestingly, CDCA treatment during early pregnancy exhibited long-term beneficial metabolic effects according to gestational age in sows.

ROS are mainly produced from mitochondrial metabolism through oxidative phosphorylation in human and mouse embryos [27]. The disturbed redox balance has been evidenced to inhibit embryo implantation through Sirt1-FoxO1-SOD signaling [28]. Furthermore, it should be noted that implantation and early pregnancy are pro-inflammatory states, as evidenced by the high levels of pro-inflammatory cytokines recorded during this phase [29]. Endometrial receptivity is the ability of the maternal endometrium to accept embryo implantation [30]. A proper inflammatory response may promote the generation of a receptive endometrium [31]; however, the inflammation must be controlled within days of conception so that decidualization and implantation can be performed [32]. For instance, TNF-α levels were lower in viable pregnancies, compared to pregnancy loss in humans [33]. Human studies also revealed similar roles for IFN-γ, IL1β, and IL6. Briefly, concentrations of the above pro-inflammatory factors were higher in patients with recurrent implantation failure [33]. In this study, our data demonstrated the beneficial effects of CDCA supplementation on governing inflammation in sows and rats during early gestation. To further corroborate these findings, there is an urgent need to understand the effects of CDCA in regulating the function of immune cells, especially T regulatory cells, which are critical for the anti-inflammatory shift that accompanies implantation and placental development [34]. Strikingly, as revealed by serum metabolomics analysis, the cortisol abundance was significantly decreased in the CDCA group. Higher plasma cortisol levels were significantly associated with the presence of metabolic syndrome, oxidative stress, and inflammation [35]. Indeed, cortisol incubation could increase the release of IL6 from undifferentiated and differentiated human mononuclear phagocytes [36]. A negative correlation between cortisol levels and antioxidant capacity was also observed in pigs [37,38]. Therefore, the decreased abundance of cortisol further suggested that CDCA supplementation alleviated inflammation and oxidative stress during early pregnancy.

Numerous studies have demonstrated that the beneficial effect of BAs is associated with their ability to increase insulin sensitivity and decrease lipid accumulation [39,40,41]. Interestingly, we observed decreased levels of glucose, TC, and fatty acids in sows of the CDCA group, implying that CDCA treatment may result in a marked enhancement in lipolysis and glucose utilization. Similar results were also observed in obese mice, where CDCA supplementation reduced glucose intolerance and dyslipidemia by inducing UCP1-mediated thermogenesis in brown adipose tissue [42]. L-carnitine transports fatty acid chains into the mitochondrial matrix, which allows cells to break down fat and derive energy from stored fat reserves. CDCA has been reported to promote *Cpt1* expression and lipid metabolism [43]. Considering the decreased lipid accumulation and fatty acid levels in the sows of the CDCA group, the possible effects of CDCA on the L-carnitine profile should be focused upon in the future. We have also previously observed deteriorated uterine receptivity and decreased implantation sites in insulin-resistant mouse models [7]. Higher maternal glucose concentrations during early pregnancy were even associated with altered fetal growth patterns and a higher risk of offspring being overweight [44,45]. As briefly mentioned above, our results suggest that CDCA administration is a potential therapeutic strategy for early gestational diabetes mellitus. Concomitant with the improved glucose and lipid metabolism, CDCA treatment also modified protein and amino acid metabolism in sows, as evidenced by the increased abundances of L-Lysine, D-Methionine, L-Methionine, L-Threonine, L-Histidine, and L-Glutamine. The beneficial effects of methionine and glutamine on embryo implantation have been well-established [10,46]. The mammalian target of rapamycin (mTOR) is a master growth regulator that senses amino acids. As reported by Chen et al., the expression levels of mTOR gradually increased from d 3 of pregnancy in the uterus of mice, reached a maximum on d 5, and then declined. Rapamycin treatment significantly decreased the number of implantation sites [47]. Therefore, more effort is needed to uncover the role of mTOR in CDCA-improved embryo implantation. 

The microbiota in the vagina, uterus, follicular fluid, and gut affects reproductive function by regulating the immune system, metabolism, hormone secretion, and other signaling pathways of the host [48]. In this study, we tested the influence of CDCA treatment during early pregnancy on gut microbiota composition and metabolism in sows. Given the easy manipulation of diets and the high level of morphological and physiological similarities in gastrointestinal systems between pigs and humans [49], the usage of pig models was also a highlight of this study. Notably, we observed obvious differences in the fecal metabolites but not in the diversity of gut microbiota between control and CDCA-treated sows. Pathway analysis showed that differential fecal metabolites were enriched in steroid hormone biosynthesis. The steroid hormones estrogen and progesterone are the main factors controlling uterine receptivity [50]. Many of the upregulated fecal metabolites identified in this study have been demonstrated to be correlated with oxidative stress, inflammation, and insulin sensitivity. For instance, N-Lac is an inhibitor of galectin-3. In a rat model of cirrhosis due to bile duct ligation, N-Lac treatment could decrease the galectin-3 and TNF-α contents in the heart [51]. The importance of sphingosine 1-phosphate (S1P)-S1P receptor signaling in inflammatory responses has also been highlighted previously [52]. More interestingly, S1P was reported to promote blastocyst formation and inhibit the embryonic cell apoptosis induced by ceramide [53]. It is unclear whether it is beneficial to embryo implantation. As a consequence of CDCA administration, the fecal levels of secondary BAs such as HDCA, LCA, isoLCA, and UDCA were also significantly increased. The negative correlations between blood glucose and HCA species were observed in humans, mice, and pigs [54]. Importantly, HDCA could improve glucose homeostasis via TGR5 and FXR signaling [54]. Additionally, gut microbiota-derived UDCA and LCA were corroborated to alleviate intestinal inflammation by inducing M2 macrophage polarization and inhibiting epithelial apoptosis in a colitis model [55]. Taken together, these altered fecal metabolites might contribute to the beneficial effects of CDCA.

A significant correlation between serum L-Histidine levels and the contents of 19 fecal metabolites suggests that L-Histidine might be a host metabolite that interacts with the gut microbiota. Histidine is an essential amino acid in mammals and has been reported to enhance antioxidant capacity in the liver through the endogenous synthesis of glutathione and NO [56]. Insulin resistance, inflammation, and oxidative stress were also attenuated in obese individuals via histidine supplementation [57]. Although the histidine load (5 g/g BW) in female rats during pregnancy and lactation could induce oxidative stress and impair energy homeostasis in the cerebral cortex and hippocampus of the offspring [58], the effects of appropriate histidine administration on embryo implantation during early pregnancy are limited. In the present study, CDCA treatment increased the serum L-Histidine levels in sows, which was associated with a decrease in the level of MDA, IFN-γ, glucose, and insulin, and an increase in the activity of SOD. Moreover, L-Histidine administration improved embryo implantation and metabolic health during early pregnancy in rats. Therefore, we propose that the beneficial effects of CDCA on embryo implantation are also related to the modulation of L-Histidine and its interaction with the gut microbiota. In the future, L-Histidine should also be administered to sows to explore its effect on embryo implantation, metabolic health, and pregnancy outcomes.

## 5. Conclusions

In summary, this study revealed that CDCA supplementation benefited embryo implantation and insulin sensitivity and alleviated inflammation and oxidative stress during early pregnancy. Mechanically speaking, in this study, CDCA regulated the metabolism of gut microbiota and increased the levels of secondary BAs. L-Histidine was identified as a critical effector contributing to the beneficial effects of CDCA during early pregnancy. These results suggest that CDCA administration is a potential therapeutic strategy against embryo loss during pregnancy.

## Figures and Tables

**Table 1:** Effects of CDCA supplementation on the body condition of sows.

Items	Control	CDCA	*p*-Value
Number of sows	12	12	-
Parity	3.78 ± 0.15	3.45 ± 0.16	0.16
Weight at breeding (kg)	221.72 ± 8.55	218.64 ± 6.91	0.78
Weight on d 14 of gestation (kg)	225.56 ± 9.78	220.45 ± 6.68	0.66
Weight on d 28 of gestation (kg)	230.06 ± 9.76	224.55 ± 7.65	0.66
Backfat thickness at breeding (mm)	15.20 ± 0.47	16.15 ± 0.43	0.16
Backfat thickness on d 14 of gestation (mm)	14.84 ± 0.63	16.20 ± 0.53	0.12
Backfat thickness on d 28 of gestation (mm)	15.18 ± 0.90	15.73 ± 0.55	0.59

**Table 2:** Effects of CDCA supplementation on the reproductive performance of sows.

Items	Control	CDCA	*p*-Value
Number of sows	12	12	-
Total piglets born per litter, *n*/litter	14.00 ± 0.93	16.73 ± 0.81	0.04
Total piglets born alive per litter, *n*/litter	12.56 ± 0.56	15.00 ± 0.85	0.04
Weight of piglets alive (kg)	1.53 ± 0.06	1.51 ± 0.07	0.84
Sex ratio (Male/Female)	1.03 ± 0.13	1.14 ± 0.23	0.71
Stillborn, n	1.11 ± 0.42	1.09 ± 0.28	0.97
Deformed, n	0.22 ± 0.22	0.27 ± 0.27	0.89
Mummified, n	0.11 ± 0.11	0.36 ± 0.20	0.32

## Data Availability

Data are contained within the article.

## Supplementary Materials

The following supporting information can be downloaded at: https://www.mdpi.com/article/10.3390/antiox13010008/s1, Table S1: Ingredients and nutrient compositions of basal diets for sows (on an as-fed basis); Table S2: Differential metabolites in the feces of sows.

## Abbreviations

ACE, abundance-based coverage estimator; BW, body weight; CAT, catalase; CDCA, chenodeoxycholic acid; d, day; DCA, deoxycholic acid; dehydroLCA, dehydrolithocholic acid; GLCA, glycolithocholic acid; GSH-Px, glutathione peroxidase; HDCA, hyodeoxycholic acid; HOMA-IR, homeostatic model assessment of insulin resistance; IFN-γ, interferon-γ; IL1β, interleukin-1β; isoLCA, isolithocholic acid; LCA, lithocholic acid; LDA, linear discriminant analysis; MDA, malondialdehyde; MDCA, murideoxycholic acid; mTOR, mammalian target of rapamycin; N-Lac, N-Acetyllactosamine; OTUs, operational taxonomic units; PCA, principal component analysis; PCoA, unweighted principal coordinate analysis; PLS-DA, partial least squares discriminant analysis; ROS, reactive oxygen species; S1P, sphingosine 1-phosphate; SOD, superoxide dismutase; T-AOC, total antioxidant capacity; TC, total cholesterol; TLCA, taurolithocholic acid; TNF-α, tumor necrosis factor-α; UDCA, ursodeoxycholic acid; VIP, variable importance projection; αMCA, alpha-muricholic acid.
