# Supplementary material for: Chenodeoxycholic Acid Improves Embryo Implantation and Metabolic Health through Modulating Gut Microbiota–Host Metabolites Interaction during Early Pregnancy
Source: Antioxidants (Basel). 2023 Dec 19;13(1):8. doi: 10.3390/antiox13010008 (PMC10812749; doi:10.3390/antiox13010008)
Supplement: Supplementary file 1 [file antioxidants-13-00008-s001.zip › antioxidants-2749136-supplementary.pdf]

Table S1. Ingredients and nutrient compositions of basal diets for sows (as-fed basis).

Items	Estrus Phase	Pregnancy Phase
Ingredients, %		
Corn	62.39	68.90
Soybean meal	18.50	14.50
Wheat bran	4.65	14.00
Fish meal	3.00	-
Extruded soybean	6.70	-
Monocalcium phosphate	0.95	0.40
Limestone	1.15	1.30
Soybean oil	1.00	-
Salt	0.30	0.40
Sodium bicarbonate	0.10	-
Sodium sulfate	0.10	-
Magnesium sulfate	0.20	-
Potassium chloride	0.15	-
L-Lysine·HCl	0.35	0.22
L-Threonine	0.05	0.04
Choline chloride (60%)	0.10	-
Phytase	-	0.02
Vitamin-mineral premix	0.31	0.23
Analyzed nutrient composition		
Total energy, MJ/kg	16.31	15.88
Crude protein, %	15.94	13.12
Amino acid composition, %		
Lysine	1.00	0.67
Methionine	0.37	0.22
Threonine	0.59	0.41
Tryptophan	0.18	0.15
Valine	0.70	0.53
Leucine	1.19	0.92
Isoleucine	0.55	0.38

Arginine	0.90	0.68
Histidine	0.38	0.29
Phenylalanine	0.75	0.58
Alanine	0.79	0.62
Aspartate	1.32	0.79
Cysteine	0.33	0.21
Glutamate	2.48	1.72
Glycine	0.66	0.49
Proline	0.85	0.70
Serine	0.68	0.47
Tyrosine	0.50	0.30
Fatty acid composition, mg/g		
C8:0	0.01	0.01
C10:0	0.02	0.02
C12:0	0.01	0.01
C14:0	0.12	0.02
C15:0	0.02	0.01
C16:0	6.13	4.16
C16:1	0.15	0.04
C17:0	0.07	0.03
C18:0	1.38	0.57
C18:1n9c	10.25	6.33
C18:2n6c	21.81	13.81
C18:3n3	1.64	0.57
C20:0	0.19	0.11
C20:1	0.13	0.11
C21:0	0.04	0.02
C20:4n6	0.03	0.02
C22:0	0.15	0.07
C20:5n3	0.22	0.02
C22:2	0.04	0.02
C23:0	0.03	0.02

C24:0	0.15	0.10
C24:1	0.03	0.00
C22:6n3	0.35	0.00

Table S2. Differential metabolites in feces of sows.

Metabolite	HMDB Superclass	HMDB Class	VIP score	Levels
Ecgonine	Alkaloids and derivatives	Tropane alkaloids	1.27	Up
Homosalate	Benzenoids	Benzene and substituted derivatives	2.25	Up
Silux	Benzenoids	Benzene and substituted derivatives	1.41	Up
Tridec-8-enedioylcarnitine	Lipids and lipid-like molecules	Fatty Acyls	2.87	Up
Sphingosine 1-phosphate (d19:1-P)	Lipids and lipid-like molecules	Sphingolipids	2.46	Up
Phorbol-12,13-dibutyrate	Lipids and lipid-like molecules	Prenol lipids	2.07	Up
LysoPC(18:2(9Z,12Z)/0:0)	Lipids and lipid-like molecules	Glycerophospholipids	1.93	Up
Cholic Acid	Lipids and lipid-like molecules	Steroids and steroid derivatives	1.79	Up
Antroquinonol	Lipids and lipid-like molecules	Prenol lipids	1.64	Up
2-Aminobenzylstatine	Organic acids and derivatives	Carboxylic acids and derivatives	2.69	Up
N-Undecanoylglycine	Organic acids and derivatives	Carboxylic acids and derivatives	1.48	Up
Arg-Thr-Lys-Arg	Organic acids and derivatives	Carboxylic acids and derivatives	1.18	Up
N-Acetyllactosamine	Organic oxygen compounds	Organooxygen compounds	3.41	Up
[3-(2-Aminopropyl)-6-methylidenecyclohexa-1,3-dien-1-yl]methanediol	Organic oxygen compounds	Organooxygen compounds	1.36	Up
Mepindolol	Organoheterocyclic compounds	Indoles and derivatives	2.58	Up
Pindolol	Organoheterocyclic compounds	Indoles and derivatives	2.29	Up
Ethoxyquin	Organoheterocyclic compounds	Quinolines and derivatives	1.84	Up
1,5-Isoquinolinediol	Organoheterocyclic compounds	Isoquinolines and derivatives	1.57	Up
1-Hydroxyisoquinoline	Organoheterocyclic compounds	Isoquinolines and derivatives	1.58	Up

Pregnan-20-one, 17-(acetyloxy)-3-hydroxy-6-methyl-, (3a,5b,6a)-	-	-	1.80	Up
N-Linoleoyl Histidine	-	-	1.39	Up
2-Hydroxyquinoline	-	-	1.08	Up
Riddelliine	Alkaloids and derivatives	-	1.52	Down
Cytochalasin B	Alkaloids and derivatives	Cytochalasans	2.02	Down
N',N'-Diphenylhydrazinecarbohydrazide	Benzenoids	Benzene and substituted derivatives	1.11	Down
3-Hydroxybenzoic Acid	Benzenoids	Benzene and substituted derivatives	1.06	Down
4-Hydroxy-5-phenyltetrahydro-1,3-oxazin-2-one	Benzenoids	Benzene and substituted derivatives	1.15	Down
Hydroxy Tyrosol -Acetate	Benzenoids	Benzene and substituted derivatives	1.28	Down
1,2-Dihydroxy-3,4-epoxy-1,2,3,4-tetrahydronaphthalene	Benzenoids	Tetralins	1.29	Down
Ketoprofen	Benzenoids	Benzene and substituted derivatives	1.12	Down
Cyclopentolate	Benzenoids	Benzene and substituted derivatives	1.59	Down
Desmethyl Fluvoxamine	Benzenoids	Benzene and substituted derivatives	1.47	Down
Gingerol	Benzenoids	Phenols	1.85	Down
Isoproterenol	Benzenoids	Phenols	2.24	Down
Pimelic Acid	Lipids and lipid-like molecules	Fatty Acyls	1.15	Down
12S-HHT	Lipids and lipid-like molecules	Fatty Acyls	1.11	Down
Demethylphylloquinone	Lipids and lipid-like molecules	Prenol lipids	1.03	Down
(2E,4E)-Hexa-2,4-dienedioylcarnitine	Lipids and lipid-like molecules	Fatty Acyls	1.11	Down
Cis-4-Decenedioic acid	Lipids and lipid-like molecules	Fatty Acyls	1.09	Down
(+)-cis-abscisic aldehyde	Lipids and lipid-like molecules	Prenol lipids	1.04	Down
(17R)-17-Hydroxy-13-methyl-17-prop-2-enyl-1,2,6,7,8,14,15,16-	Lipids and lipid-like molecules	Steroids and steroid derivatives	1.10	Down

octahydrocyclopenta[a]phenanthren-3-one				
Estriol	Lipids and lipid-like molecules	Steroids and steroid derivatives	1.16	Down
5-Acetamidovalerate	Lipids and lipid-like molecules	Fatty Acyls	1.19	Down
Pantothenol	Lipids and lipid-like molecules	Fatty Acyls	1.04	Down
Doisynoestrol	Lipids and lipid-like molecules	Steroids and steroid derivatives	1.04	Down
Glycerylphosphorylcholine	Lipids and lipid-like molecules	Glycerophospholipids	1.11	Down
3-Hydroxy-cis-5-octenoylcarnitine	Lipids and lipid-like molecules	Fatty Acyls	1.09	Down
Oxymesterone	Lipids and lipid-like molecules	Steroids and steroid derivatives	1.05	Down
28-Homobrassinolide	Lipids and lipid-like molecules	Steroids and steroid derivatives	1.22	Down
Genipin	Lipids and lipid-like molecules	Prenol lipids	1.18	Down
Prostaglandin I2	Lipids and lipid-like molecules	Fatty Acyls	1.38	Down
Ethyl (S)-3-hydroxybutyrate glucoside	Lipids and lipid-like molecules	Fatty Acyls	1.24	Down
Prednisone acetate	Lipids and lipid-like molecules	Steroids and steroid derivatives	1.37	Down
5-Hydroxyvalproic acid	Lipids and lipid-like molecules	Fatty Acyls	1.16	Down
Glycerophosphocholine	Lipids and lipid-like molecules	Glycerophospholipids	1.33	Down
Hexadecanedioic acid	Lipids and lipid-like molecules	Fatty Acyls	1.45	Down
(5S,6R)-Methyl 5,6,7-trihydroxyheptanoate	Lipids and lipid-like molecules	Fatty Acyls	1.36	Down
Butyl (S)-3-hydroxybutyrate glucoside	Lipids and lipid-like molecules	Fatty Acyls	1.60	Down
4-Deacetylneosolaniol	Lipids and lipid-like molecules	Prenol lipids	1.32	Down
Octa-3,6-dienedioylcarnitine	Lipids and lipid-like molecules	Fatty Acyls	1.53	Down
Artecanin	Lipids and lipid-like molecules	Prenol lipids	1.31	Down
Goshuyic acid	Lipids and lipid-like molecules	Fatty Acyls	1.58	Down

Delta-Tocopherol	Lipids and lipid-like molecules	Prenol lipids	1.49	Down
Aldosterone	Lipids and lipid-like molecules	Steroids and steroid derivatives	1.52	Down
Jacarandic acid	Lipids and lipid-like molecules	Prenol lipids	1.32	Down
21-Deoxycortisol	Lipids and lipid-like molecules	Steroids and steroid derivatives	1.60	Down
4alpha-carboxy-5alpha-cholesta-8,24-dien-3beta-ol	Lipids and lipid-like molecules	Steroids and steroid derivatives	1.18	Down
Prostaglandin J2	Lipids and lipid-like molecules	Fatty Acyls	1.57	Down
Ingenol	Lipids and lipid-like molecules	Prenol lipids	1.65	Down
2-Ethyl-2-hydroxybutanedioylcarnitine	Lipids and lipid-like molecules	Fatty Acyls	1.36	Down
Prostaglandin A2	Lipids and lipid-like molecules	Fatty Acyls	1.69	Down
21-Deoxycortisone	Lipids and lipid-like molecules	Steroids and steroid derivatives	1.66	Down
Heptanoic acid	Lipids and lipid-like molecules	Fatty Acyls	1.57	Down
Isopropylmaleic acid	Lipids and lipid-like molecules	Fatty Acyls	1.63	Down
Demissidine	Lipids and lipid-like molecules	Steroids and steroid derivatives	1.99	Down
2-Hydroxyestradiol	Lipids and lipid-like molecules	Steroids and steroid derivatives	1.86	Down
3-Hydroxybutyrylcarnitine	Lipids and lipid-like molecules	Fatty Acyls	2.14	Down
Boldenone	Lipids and lipid-like molecules	Steroids and steroid derivatives	2.47	Down
7-Methyl-2'-deoxyguanosine-3'-monophosphate	Nucleosides, nucleotides, and analogues	Ribonucleoside 3"-phosphates	1.61	Down
Phenylalanylproline	Organic acids and derivatives	Carboxylic acids and derivatives	1.09	Down
((4-(4-Amidinophenoxy)butanoyl)aspartyl)valine	Organic acids and derivatives	Carboxylic acids and derivatives	1.01	Down
Clavulanate	Organic acids and derivatives	Carboxylic acids and derivatives	1.10	Down
N-(1-Carboxy-3-carboxanilidopropyl)alanylproline	Organic acids and derivatives	Carboxylic acids and derivatives	1.10	Down
N-(2,3,4-Trihydroxybutyl)-L-valine	Organic acids and derivatives	Carboxylic acids and derivatives	1.13	Down

Glycylproline	Organic acids and derivatives	Carboxylic acids and derivatives	1.12	Down
Arginyltryptophan	Organic acids and derivatives	Carboxylic acids and derivatives	1.28	Down
L-Theanine	Organic acids and derivatives	Carboxylic acids and derivatives	1.36	Down
Methylene bisacrylamide	Organic acids and derivatives	Carboxylic acids and derivatives	1.24	Down
Domoic acid	Organic acids and derivatives	Carboxylic acids and derivatives	1.37	Down
1-Pyrroline-5-carboxylic acid	Organic acids and derivatives	Carboxylic acids and derivatives	1.18	Down
Homocitrulline	Organic acids and derivatives	Carboxylic acids and derivatives	1.20	Down
Enalaprilat	Organic acids and derivatives	Carboxylic acids and derivatives	1.42	Down
Indolylacryloylglycine	Organic acids and derivatives	Carboxylic acids and derivatives	1.14	Down
Deferitrin	Organic acids and derivatives	Carboxylic acids and derivatives	1.33	Down
5-Keto-D-Gluconate	Organic acids and derivatives	Hydroxy acids and derivatives	1.23	Down
Enbucrilate	Organic acids and derivatives	Carboxylic acids and derivatives	1.49	Down
2-[4-(Carboxymethyl)-1,4,8,11-tetrazabicyclo[6.6.2]hexadecan-11-yl]acetic acid	Organic acids and derivatives	Carboxylic acids and derivatives	1.55	Down
D-1-[(3-Carboxypropyl)amino]-1-deoxyfructose	Organic acids and derivatives	Carboxylic acids and derivatives	1.56	Down
Fructosylvaline	Organic acids and derivatives	Carboxylic acids and derivatives	1.53	Down
Frangulanine	Organic acids and derivatives	Carboxylic acids and derivatives	1.48	Down
MeOSuc-Ala-Ala-Pro-Val-PNA	Organic acids and derivatives	Carboxylic acids and derivatives	1.78	Down
N2-Acetylornithine	Organic acids and derivatives	Carboxylic acids and derivatives	1.51	Down
6-Guanidino-2-oxocaproic acid	Organic acids and derivatives	Keto acids and derivatives	1.45	Down
Melilotocarpan A	Organic acids and derivatives	Carboxylic acids and derivatives	1.58	Down
Cyclopropanecarboxylic acid	Organic acids and derivatives	Carboxylic acids and derivatives	1.67	Down

N-Palmitoyl Leucine	Organic acids and derivatives	Carboxylic acids and derivatives	1.97	Down
S-(N,N-Diethylcarbamoyl)glutathione	Organic acids and derivatives	Carboxylic acids and derivatives	1.77	Down
N-Stearoyl Leucine	Organic acids and derivatives	Carboxylic acids and derivatives	1.88	Down
(1R,6R)-6-hydroxy-2-succinylcyclohexa-2,4-diene-1-carboxylate	Organic acids and derivatives	Keto acids and derivatives	2.12	Down
Seglitide	Organic acids and derivatives	Carboxylic acids and derivatives	2.45	Down
Pyridinoline	Organic acids and derivatives	Carboxylic acids and derivatives	1.84	Down
Lyciumin B	Organic acids and derivatives	Carboxylic acids and derivatives	3.00	Down
Oxytocin 1-8	Organic acids and derivatives	Carboxylic acids and derivatives	3.29	Down
Perindopril Acyl-beta-D-glucuronide	Organic acids and derivatives	Carboxylic acids and derivatives	3.43	Down
Isokobusone	Organic oxygen compounds	Organooxygen compounds	1.10	Down
THYMOQUINONE	Organic oxygen compounds	Organooxygen compounds	1.36	Down
Isopropyl beta-D-glucoside	Organic oxygen compounds	Organooxygen compounds	1.42	Down
TRIAZIQUONE	Organic oxygen compounds	Organooxygen compounds	1.31	Down
Alliospiroside D	Organic oxygen compounds	Organooxygen compounds	2.28	Down
Eutypine	Organic oxygen compounds	Organooxygen compounds	2.86	Down
D-Biotin	Organoheterocyclic compounds	Biotin and derivatives	1.04	Down
Uracil	Organoheterocyclic compounds	Diazines	1.07	Down
(S)-N-Methylcoclaurine	Organoheterocyclic compounds	Isoquinolines and derivatives	1.10	Down
Spectinomycin	Organoheterocyclic compounds	Dioxanes	1.16	Down
Oxypurinol	Organoheterocyclic compounds	Imidazopyrimidines	1.31	Down
Monocrotaline	Organoheterocyclic compounds	Pyrrolizines	1.31	Down
Tert-butyl 4-(1H-pyrazolo[3,4-d]pyrimidin-4-yl)piperazine-1-	Organoheterocyclic compounds	Diazinanes	1.27	Down

carboxylate				
VASICINONE	Organoheterocyclic compounds	Diazanaphthalenes	1.15	Down
N-Acetylserotonin	Organoheterocyclic compounds	Indoles and derivatives	1.25	Down
Hydroxymethyl cimetidine	Organoheterocyclic compounds	Azoles	1.31	Down
Pi-Methylimidazoleacetic acid	Organoheterocyclic compounds	Azoles	1.21	Down
7-Aminomethyl-7-carbaguanine	Organoheterocyclic compounds	Pyrrolopyrimidines	1.37	Down
Methyl 5-hydroxyoxindole-3-acetate	Organoheterocyclic compounds	Indoles and derivatives	1.50	Down
7-Methylhypoxanthine	Organoheterocyclic compounds	Imidazopyrimidines	1.39	Down
Petasitenine	Organoheterocyclic compounds	Azaspirodecane derivatives	1.57	Down
Neopterin	Organoheterocyclic compounds	Pteridines and derivatives	1.46	Down
Citrinin	Organoheterocyclic compounds	Benzopyrans	1.62	Down
6-hydroxymethyl-7,8-dihydropterin	Organoheterocyclic compounds	Pteridines and derivatives	1.26	Down
Petasinine	Organoheterocyclic compounds	Pyrrolizidines	1.66	Down
3h-Serotonin	Organoheterocyclic compounds	Indoles and derivatives	1.82	Down
Citreoviridin	Organoheterocyclic compounds	Pyrans	1.61	Down
(+/-)-Ribaline	Organoheterocyclic compounds	Quinolines and derivatives	2.40	Down
Velpatasvir	Organoheterocyclic compounds	Naphthopyrans	2.42	Down
1-Aminoacridine	Organoheterocyclic compounds	Quinolines and derivatives	2.16	Down
Methylisopelletierine	Organoheterocyclic compounds	Piperidines	2.36	Down
25-O-Desacetyl rifabutin	Organoheterocyclic compounds	Naphthofurans	3.05	Down
Methylimidazoleacetic acid	Organoheterocyclic compounds	Azoles	2.55	Down
N-[(2R,3R,4S,6R)-4,6-Dihydroxy-6-methyl-2-[(1R,2R)-1,2,3-	Organoheterocyclic compounds	Oxanes	3.03	Down

trihydroxypropyl]oxan-3-yl]acetamide				
Negletein 6-[rhamnosyl-(1->2)-fucoside]	Organoheterocyclic compounds	Benzopyrans	2.12	Down
Cowanin	Organoheterocyclic compounds	Benzopyrans	2.39	Down
Evobioside	Organoheterocyclic compounds	Furans	3.08	Down
Alpha-Methyl-m-tyrosine	Phenylpropanoids and polyketides	Phenylpropanoic acids	1.03	Down
Neobyakangelicol	Phenylpropanoids and polyketides	Coumarins and derivatives	1.03	Down
Milbemycin beta1	Phenylpropanoids and polyketides	Macrolides and analogues	1.07	Down
Sayanedin	Phenylpropanoids and polyketides	Isoflavonoids	1.15	Down
Cajanol	Phenylpropanoids and polyketides	Isoflavonoids	1.12	Down
Phellopterin	Phenylpropanoids and polyketides	Coumarins and derivatives	1.15	Down
Cinanserin	Phenylpropanoids and polyketides	Cinnamic acids and derivatives	1.36	Down
Xenognosin A	Phenylpropanoids and polyketides	Linear 1,3-diarylpropanoids	1.21	Down
(S,E)-Zearalenone	Phenylpropanoids and polyketides	Macrolides and analogues	1.44	Down
Saxitoxin	Phenylpropanoids and polyketides	Saxitoxins, gonyautoxins, and derivatives	1.39	Down
4-coumaroylshikimate	Phenylpropanoids and polyketides	Cinnamic acids and derivatives	1.51	Down
Caffeic Acid	Phenylpropanoids and polyketides	Cinnamic acids and derivatives	1.20	Down
Homopisatin	Phenylpropanoids and polyketides	Isoflavonoids	1.47	Down
Isoferulic acid	Phenylpropanoids and polyketides	Cinnamic acids and derivatives	1.53	Down
Vestitone	Phenylpropanoids and polyketides	Isoflavonoids	1.56	Down
Diferuloylputrescine	Phenylpropanoids and polyketides	Cinnamic acids and derivatives	2.56	Down
Novobiocin	Phenylpropanoids and polyketides	Coumarins and derivatives	3.23	Down
6-Maleimidocaproic acid	-	-	1.04	Down

Lauryldiethanolamine	-	-	1.19	Down
Citrazinic Acid	-	-	1.32	Down
Asn Asp Lys	-	-	1.45	Down
Elexacaftor/Ivacaftor/Tezacaftor	-	-	2.19	Down
Thr Asn Tyr	-	-	2.36	Down
PGP(16:1(9Z)/18:3(10,12,15)-OH(9))	-	-	3.42	Down
